# A Novel Automated System Yields Reproducible Temporal Feeding Patterns in Laboratory Rodents

**DOI:** 10.1093/jn/nxz116

**Published:** 2019-07-09

**Authors:** Thomas W Tilston, Richard D Brown, Matthew J Wateridge, Bradley Arms-Williams, Jamie J Walker, Yuxiang Sun, Timothy Wells

**Affiliations:** 1 Neuroscience and Mental Health Research Institute and School of Biosciences, Cardiff University, Cardiff, United Kingdom; 2 College of Engineering, Mathematics and Physical Sciences, University of Exeter, Exeter, United Kingdom; 3 Wellcome Trust Centre for Biomedical Modelling and Analysis, University of Exeter, Exeter, United Kingdom; 4 EPSRC Centre for Predictive Modelling in Healthcare, University of Exeter, Exeter, United Kingdom; 5 Henry Wellcome Laboratories for Integrative Neuroscience and Endocrinology, University of Bristol, Bristol, United Kingdom; 6 Department of Nutrition and Food Science, Texas A&M University, College Station, TX, USA

**Keywords:** feeding patterns, meal-feeding, grazing, meal microstructure, automated feeding, automated blood sampling, corticosterone profiles, ghrelin

## Abstract

**Background:**

The impact of temporal feeding patterns remains a major unanswered question in nutritional science. Progress has been hampered by the absence of a reliable method to impose temporal feeding in laboratory rodents, without the confounding influence of food-hoarding behavior.

**Objective:**

The aim of this study was to develop and validate a reliable method for supplying crushed diets to laboratory rodents in consistent, relevant feeding patterns for prolonged periods.

**Methods:**

We programmed our experimental feeding station to deliver a standard diet [StD; Atwater Fuel Energy (AFE) 13.9% fat] or high-fat diet (HFD; AFE 45% fat) during nocturnal grazing [providing 1/24th of the total daily food intake (tdF/I) of ad libitum–fed controls every 30 min] and meal-fed (3 × 1-h periods of ad libitum feeding) patterns in male rats (Sprague-Dawley: 4 wk old, 72–119 g) and mice [C57/Bl6J wild-type (WT): 6 mo old, 29–37 g], and *ghrelin*-null littermates (Ghr^−/−^; 27–34 g).

**Results:**

Grazing yielded accurate, consistent feeding events in rats, with an approximately linear rise in nocturnal cumulative food intake [tdF/I (StD): 97.4 ± 1.5% accurate compared with manual measurement; *R^2^* = 0.86; tdF/I (HFD): 99.0 ± 1.4% accurate; *R^2^* = 0.86]. Meal-feeding produced 3 nocturnal meals of equal size and duration in StD-fed rats (tdF/I: 97.4 ± 0.9% accurate; *R^2^* = 0.90), whereas the second meal size increased progressively in HFD-fed rats (44% higher on day 35 than on day 14; *P* < 0.01). Importantly, cumulative food intake in grazing and meal-fed rats was identical. Similar results were obtained in WT mice except that less restricted grazing induced hyperphagia (compared with meal-fed WT mice; *P* < 0.05 from day 1). This difference was abolished in Ghr^−/−^ mice, with meal initiation delayed and meal duration enhanced. Neither pattern elevated corticosterone secretion in rats, but meal-feeding aligned ultradian pulses.

**Conclusions:**

We have established a consistent, measurable, researcher-defined, stress-free method for imposing temporal feeding patterns in rats and mice. This approach will facilitate progress in understanding the physiologic impact of feeding patterns.

## Introduction

The impact of temporal feeding patterns remains a major unanswered question in nutritional science (1, 2). Many epidemiologic studies indicate that human feeding patterns are associated with physiologic variables such as food choice (3), energy intake (4), and metabolic outcome (5, 6), but not all agree (7). In addition to their inability to confirm causation, the conclusions of these studies are compromised by the unreliability of self-reported food intake (8, 9) and the potential distortion of participant attrition (10). The development of mobile apps has partially alleviated this problem (11, 12), but reliable evidence can only be obtained from intervention studies in a fully controlled environment. Although equivalence in humans will need to be established, such studies are most expeditiously performed in laboratory animals, this context providing the added advantage that the existence of a wide range of genetically modified models enables rapid establishment of potential mechanisms.

Delivering relevant, reproducible feeding patterns to laboratory rodents over sustained periods has been hampered by technical limitations, with many published chrononutritional studies being restricted to relatively crude manipulations, such as reversed feeding (13, 14), caloric restriction (15, 16), and single time-point feeding occasions (17, 18). Although the findings of these studies largely support the epidemiologic data, they fail to replicate contemporary human feeding patterns and are compromised by confounding factors such as the preobesogenic influence of light phase–only feeding (14) and the pleiotropic consequences of food restriction (16).

A number of approaches have been taken to tackle this problem (19). These include the ClockLab (20), BioDAQ (21), and SnackClock (22) systems, which permit rodents to receive pelleted food in user-defined patterns. However, monitoring pellet loss from the hopper does not necessarily equate with food intake because supplying pelleted diet enables rodents to hoard food in the home cage, a behavior especially prominent in female mice (23, 24). The potential presence of a hoarded store permits uncontrolled feeding and compromises confidence in the physiologic endpoints measured. In addition, supplying pelleted diet also precludes delivering the smoothed food access required to study snacking/grazing behavior.

We have taken a more sophisticated approach to replicating relevant human feeding patterns in laboratory rodents. Modified Oxymax control software for the Columbus Instruments Comprehensive Laboratory Animal Monitoring System (CLAMS) has enabled the programming of multiple feeding events in which access to crushed diet can be regulated by time and the amount consumed (**Supplemental Figure 1**). We have used this approach to deliver 2 specific nocturnal feeding patterns—grazing and meal feeding—in rats and mice, and have used automated serial blood sampling to monitor the effects of these feeding patterns on concurrent hormone secretion. Given the well-established role of ghrelin (Ghr) in meal initiation (25, 26) and that the biological influence of Ghr is dependent upon the pattern of exposure (27, 28), we have also characterized the effects of these feeding patterns in *ghrelin*-null (Ghr^−/−^) mice.

## Methods

### Animals

The animal procedures reported here (including those involving genetically modified mice) were performed under the authority of the Animals (Scientific Procedures) Act, 1986 (UK) in accordance with the ARRIVE guidelines and were specifically approved by local ethical review. Male Sprague-Dawley rats (studies 1, 2, and 3) were purchased from Charles River and housed immediately upon receipt in the system described below. Male C57/Bl6J wild-type (WT) and homozygous Ghr^−/−^ littermate mice (study 4) were obtained from heterozygous × heterozygous matings of breeding stock derived from embryos imported from the vivarium at Baylor College of Medicine, with the genotype identified by PCR analysis of DNA extracted from ear punches, as previously described (29). All experimental animals were housed in individual cages (as detailed below) in the metabolic room of the JBIOS animal facility, Cardiff University under conditions of 12 h light/12 h dark (lights on at 0600), with water available ad libitum and diet supplied as described in detail below.

### CLAMS system

The CLAMS system at Cardiff University consists of 4 individual rat cages (Supplemental Figure 1A), in which access to the underfloor food hopper is regulated by a pneumatic servocontrolled lid (Supplemental Figure 1A, B). Each hopper is located on an electronic balance, and its weight is recorded by Oxymax control software (Oxymax for Windows version 4.44 and later) every 90 s. These software versions include a modification enabling the programming of multiple individual food access events during each 24-h period. By setting the system to permit dietary access to be regulated by time and the amount of food consumed, we were able to establish the dietary patterns described below.

#### Nocturnal grazing

Grazing rats were permitted access to 1/24th of the mean total daily food intake (tdF/I) of a concurrent cohort of 3 age-matched ad libitum–fed control rats every 30 min during the dark phase, with the first access period timed to coincide with lights out (1800). When this amount had been consumed, the lid closed for the remainder of the 30-min period. As the daily food intake of the growing ad libitum–fed rats increased, the allowance supplied to grazing rats was increased in parallel. Thus, grazing rats were not permitted to consume any large meals (Supplemental Figure 1D).

#### Nocturnal meal-feeding

Meal-fed rats were permitted three 1-h periods of ad libitum access to the diet at the beginning (1800) middle (2330) and end (0500) of the dark phase, the access lid remaining closed at all other times. Thus, meal-fed rats were not permitted to graze between meals (Supplemental Figure 1E).

#### Ad libitum feeding

In order to calculate the food intake allowance for grazing rats a cohort of age- and weight-matched animals (≥3 per experimental cohort) housed in either standard transparent cages (rats; catalog no. 2154, Tecniplast UK Ltd) or metabolic cages (mice; catalog no. 3700M061; Tecniplast UK Ltd) and permitted ad libitum access to the same crushed diet (see below for dietary details), with daily consumption quantified between 0900 and 1000 h (Supplemental Figure 1C shows a representative intake profile in rats).

### Procedural considerations

The CLAMS cages are supplied with a stainless-steel hood over the food access lid. This was left in position for the first week of each study to train the animals to approach the hopper from the front of the cage (where the lid opening is widest). However, the hood was removed thereafter to prevent restricting access for growing rats and those prepared with indwelling catheters, and to prevent mice from nesting under the hood. In addition, because rats are very adaptable and will utilize any movable objects to maintain the food access lid in an open position, items supplied for environmental enrichment were restricted to immovable objects.

### Study 1: Delivering a standard diet in either grazing or meals in male rats

Three groups of 4-wk-old male Sprague-Dawley rats (weighing 83.8–118.8 g) consumed a standard nonpurified rodent diet (StD) [SDS RM3; Special Diet Services Ltd; Atwater Fuel Energy (AFE) 13.9% fat; full dietary components of all diets are listed in **Supplemental Table 1**] in either ad libitum, grazing, or meal-fed patterns for 6 wk. Scanned output from the undercage balances was stored and used to calculate feeding events and cumulative food consumption (grazing and meal-fed rats only) with daily cumulative food consumption compared with data obtained by daily weighing of food hoppers. Given the amount of data generated (40,320 event values for each animal), we selected data from days 14 and 35 for more detailed analysis of pattern consistency and feeding microstructure.

### Study 2: Delivering a high-fat diet in either grazing or meals in male rats

Four groups of 4-wk-old male Sprague-Dawley rats (weighing 71.6–111.9 g) consumed either a low-fat diet (LFD) (SDS 824040; Special Diet Services Ltd; AFE 10% fat) or high-fat diet (HFD) (SDS 824043: Special Diet Services Ltd; AFE 45% fat) ad libitum, or an HFD in either a grazing or meal-fed pattern for 6 wk. Food intake was quantified daily and scanned output data were processed as in study 1 above.

### Study 3: Do these meal patterns activate the stress axis?

In order to investigate whether the application of grazing and meal-feeding activated the hypothalamopituitary adrenal (HPA) axis, 6-wk-old male Sprague-Dawley rats (weighing 104.3–128.0 g) were individually housed in either metabolic cages (catalog no. 3700M071; Tecniplast UK Ltd) and provided with StD ad libitum, or in CLAMS cages for grazing or meal-feeding (as in study 1) for 3 wk. On day 18 each rat was prepared with a single-bore jugular vein catheter (0.5 mm internal diameter × 40.7 cm length; 100 μL internal volume) under isoflurane anesthesia, the catheter being exteriorized through the scalp and anchored in place with a protective spring which was secured above the cage to a single-bore swivel (Supplemental Figure 1A, B). Rats were permitted 48 h for recovery, during which food delivery patterns were continued and food intake and body weights monitored daily. Each catheter was connected via a swivel to an automated serial blood sampling system [manufactured at Bristol University, UK based on a system designed at the National Institute for Medical Research, London (30)] primed with sterile heparinized saline (10 IU/mL). Catheter patency was maintained prior to blood sampling with an intermittent catheter flushing protocol, in which blood was drawn to the top of each catheter every hour and returned with the additional infusion of a 20-μL bolus of heparinized saline.

Automated serial blood sampling was commenced at the beginning of the light phase (0600) on day 20, and involved collecting 100 μL of 1:5 blood (20 μL blood in 80 μL heparinized saline) every 10 min for 24 h. Blood samples were collected into microtiter tubes in 96-well format blocks on a refrigerated fraction collector, vortexed, centrifuged at 2773 × *g*; 10 min; 4°C, and 25-μL subsamples of diluted plasma were collected, freeze-dried, and stored at −20°C for subsequent quantification of corticosterone by radioimmunoassay (see below).

On day 21 rats were reanesthetized with isoflurane and decapitated. Adrenal glands were dissected and weighed.

### Study 4: Can grazing and meal-feeding protocols be used in mice and are the effects seen Ghr dependent?

Three groups of 6-mo-old male WT mice [29.4–37.4 g; mean ± SEM 33.1 ± 0.45 (*n* = 18)] and 3 groups of male Ghr^−/−^ littermates [28.4–33.9 g; mean ± SEM 30.9 ± 0.4 (*n* = 18); *P* < 0.001] were housed in either metabolic cages (catalog no. 3700M061; Tecniplast UK Ltd) and consumed StD ad libitum, or in CLAMS cages for grazing or meal feeding for 3 wk. The meal-feeding protocol was conducted as in study 1, but, given their age, grazing mice were provided with a set amount of diet (0.5 g) every 30 min during the dark phase for the whole duration of the study (i.e., not pair-fed with ad libitum–fed controls).

Food intake and body weight were monitored daily throughout the experiment, with scanned output data for day 14 from grazing and meal-fed mice processed as in study 1 above. On day 21 mice were anesthetized with isoflurane and decapitated. Adrenal glands were dissected and weighed.

### Radioimmunoassay for corticosterone

The plasma corticosterone concentrations in samples from study 3 were determined by radioimmunoassay, as previously described (31, 32). In brief, freeze-dried samples were reconstituted in 25 μL deionized water, acidified in a sodium citrate buffer (pH 3.0) to denature corticosterone-binding globulin, and a competitive binding assay conducted with a specific rabbit anti-rat corticosterone primary antibody (generously supplied by Dr Dóra Zelena, Institute of Experimental Medicine, Budapest, Hungary) and ^125^I-labeled rat corticosterone (IRC-123; Institute of Isotopes Co. Ltd, Budapest, Hungary). The assay sensitivity range was 0.98–2000 ng/mL, with intra- and interassay variations of 3.25% and 16.53%, respectively.

### Statistical analyses

Feeding profiles are presented from either individual animals or as mean data from similarly fed animals. Daily feeding profiles show individual feeding events with the superimposition of corresponding cumulative food intake data or corresponding hormone profiles. Meal duration was determined by the last time point at which ≥0.02 g diet was consumed. Regression analysis (Microsoft Excel version 16.15 for Mac) was used to assess the accuracy of the CLAMS data output.

Total hormone secretory output was determined by calculation of the AUC (Microsoft Excel version 16.15 for Mac). Distribution analysis was used to calculate the “observed concentration 5” (OC_5_; the cutoff value below which 5% of the samples fall when ranked in ascending order of concentration) as an index of baseline secretion (33). To determine parameters of circadian secretion, a fixed 24-h period sine function was fitted to individual hormone profiles in the least-squares sense (see Supplemental Figure 6). The circadian peak was taken as the time corresponding to the maximum in the fitted sine function. The parameters of ultradian secretion were determined from the individual hormone profiles by cluster analysis (34), a statistically rigorous peak-detection algorithm that has been widely used to quantify pulsatile hormone dynamics. The algorithm detected statistically significant corticosterone pulses, pulse frequency (pulses/h), and pulse height (ng/mL) in each profile. The cluster parameters used in the analysis were as follows: test cluster size for sliding nadir, 2.0; test cluster size for sliding peak, 1.0; *t* statistic for significant increase in the data, 2.0; *t* statistic for significant decrease in the data, 2.0; and minimum peak size, 0.0 ng/mL.

All statistical analyses were performed on data from individual animals and the group data are presented as means ± SEMs. Comparisons of time-dependent parameters within each group were made by paired Student's *t* test and comparisons between different groups by either an unpaired Student's *t* test (Microsoft Excel version 16.15 for Mac) or 1-factor ANOVA and either Bonferroni post-hoc test or Kruskal-Wallis and Dunn's multiple comparison test (GraphPad Prism, version 7.0d for Mac OS X) as indicated in the figure and table legends, with *P* < 0.05 being considered significantly different.

## Results

### Study 1: Delivering a standard diet in either grazing or meals in male rats

**FIGURE 1:**
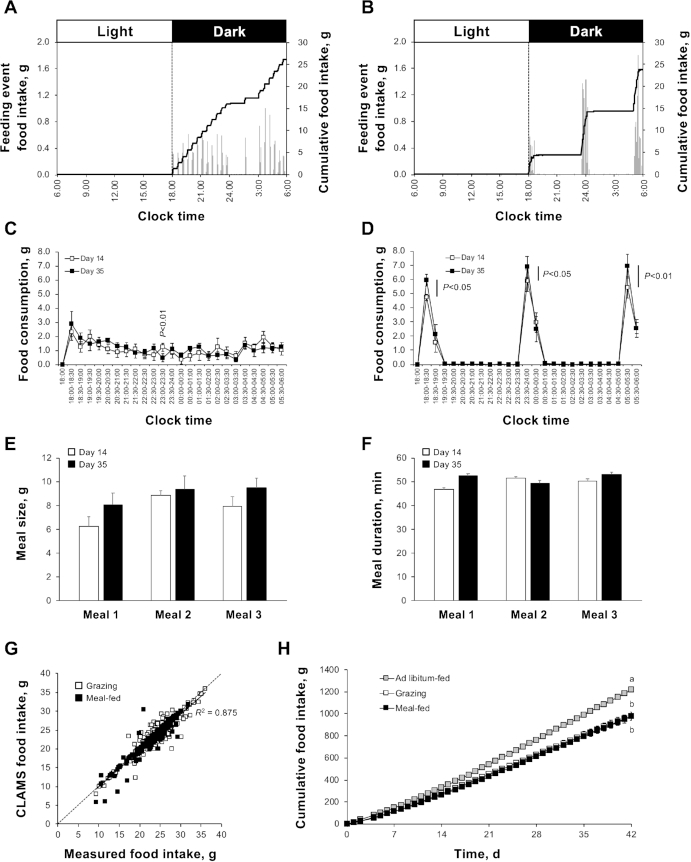
Delivery of StD in standard grazing and meal-feeding patterns in male rats (study 1). Representative daily food intake profiles from day 14 of a 6-wk study in grazing (A) and meal-fed (B) rats consuming StD (13.9% Atwater Fuel Energy fat). Each profile shows cumulative food intake (black line; right-hand axes) superimposed onto the individual feeding events (gray bars; left-hand axes). Comparison of the mean day-14 and day-35 food consumption profiles in grazing (C) and meal-fed (D) rats is shown, with quantification of the size (E) and duration (F) of the individual meals in meal-fed animals. Regression analysis of the Comprehensive Laboratory Animal Monitoring System output data with manual quantification of daily food consumption is shown (G; regression conducted for all data points; dashed line shows parity), with cumulative food consumption across the 6-wk study presented (H). Apart from the representative individual profiles (A, B), data shown are means ± SEMs [C–F, H; *n* = 6 (grazing) and 5 (meal-fed)] with statistical comparisons performed by either paired Student's *t* test (C, D) or 1-factor ANOVA and Bonferroni's selected-pairs post-hoc test (E, F, H) (means without a common letter differ, *P* < 0.05). Note: a group of ad libitum–fed rats (H; gray symbols; *n* = 8) was monitored in parallel to calculate food intake allowance for application to grazing rats. StD, standard nonpurified rodent diet.

The effectiveness of our automated feeding station was assessed by programming it to deliver powdered StD (AFE 13.9% fat) in either grazing or meal-fed patterns (Supplemental Figure 1). Representative and mean profiles (**Figure 1**A, C) indicate that our nocturnal grazing protocol (rats receiving 1/24th of the tdF/I of ad libitum–fed controls every 30 min) produced an approximately linear rise in cumulative food intake across the dark phase. Although individual animals may show periods of reduced feeding (e.g., from 2400 to 0300 in Figure 1A), the overall pattern of intake was maintained throughout the study (Figure 1C). Analysis of feeding period microstructure from the individual 90 second-balance scans revealed that only 10 of the 480 time points were significantly different between days 14 and 35 (**Supplemental Figure 2**; *P* < 0.05).

Meal-fed rats received three 1-h periods of ad libitum food access at the beginning, middle, and end of the dark phase. This resulted in the delivery of 3 evenly spaced meals of equal size (Figure 1E) and duration (Figure 1F), producing a ramped increase in cumulative food intake (Figure 1B). Although total meal size had not increased by day 35, diet consumption during the first 30 min of each meal increased by 26%, 16%, and 27%, respectively (Figure 1D; *P* < 0.05). Analysis of meal microstructure revealed that only 8 of the 480 time points were different between days 14 and 35 (**Supplemental Figure 3**; *P* < 0.05).

There was a strong correlation between the CLAMS output data and data derived from manual measurement of daily food intake (*R*^2^ = 0.88, both patterns; Figure 1G). This corresponded to a mean accuracy for individual rats of 97.4% ± 0.9% (all days). Similar relations were observed in grazing-only (*R*^2^ = 0.86; accuracy: 97.4% ± 1.5%) and meal-fed-only (*R*^2^ = 0.90; accuracy: 97.4% ± 0.9%) data.

Interestingly, in comparison with the ad libitum–fed controls, grazing and meal-fed rats showed a 20% reduction in total cumulative food intake (*P* < 0.0001; Figure 1H), which was apparent from day 1 onwards. Importantly, grazing and meal-fed rats consumed the same quantity of diet (Figure 1H).

### Study 2: Delivering a high-fat diet in either grazing or meals in male rats

To determine whether this system was equally effective in delivering alternative diets, we provided crushed HFD (AFE 45% fat) in the grazing and meal-fed patterns used in study 1. In addition to the rats consuming the HFD ad libitum, a further group of rats consumed an LFD (AFE 10% fat) ad libitum.

**FIGURE 2:**
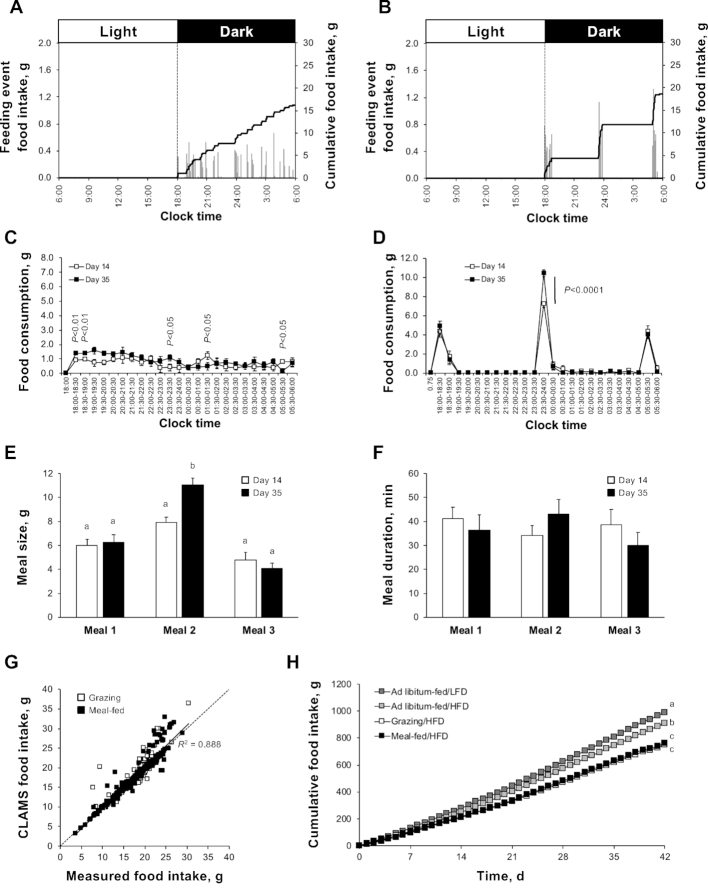
Delivery of HFD in grazing and meal-feeding patterns in male rats (study 2). Representative daily food intake profiles from day 14 of a 6-wk study in grazing (A) and meal-fed (B) rats consuming HFD (45% Atwater Fuel Energy fat). Each profile shows cumulative food intake (black line; right-hand axes) superimposed onto the individual feeding events (gray bars; left-hand axes). Comparison of the mean day-14 and day-35 food consumption profiles in grazing (C) and meal-fed (D) rats is shown, with quantification of the size (E) and duration (F) of the individual meals in meal-fed animals. Regression analysis of the Comprehensive Laboratory Animal Monitoring System output data with manual quantification of daily food consumption is shown (G; regression conducted for all data points; dashed line shows parity), with cumulative food consumption across the 6-wk study presented (H). Data shown are either representative individual profiles (A, B), or means ± SEMs [C–F, H; *n* = 6 (grazing and meal-fed)] with statistical comparisons performed by either paired Student's *t* test (C, D) or 1-factor ANOVA and Bonferroni's selected-pairs post-hoc test (E, F, H) (means without a common letter differ, *P* < 0.05). Note: groups of rats consuming an HFD or low-fat diet ad libitum (H; *n* = 9 both diets) were monitored in parallel to calculate food intake allowance for application to grazing rats. HFD, high-fat diet; LFD, low-fat diet.

Rats grazing on the HFD showed an approximately linear rise in cumulative food intake across the dark phase (**Figure 2**A, C), which was sustained throughout the study. Daily food intake increased by 26% from day 14 to day 35 (*P* < 0.05), which was almost entirely due to an increase in food intake in the first quarter of the dark phase (Figure 2C). Microstructure analysis (**Supplemental Figure 4**) revealed that animals grazing on the HFD exhibited rapid consumption of the half-hourly allowance in the first 9 min of each 30-min period (c.f. Supplemental Figure 2). This phenomenon, which was especially prominent on day 35, subsided as the dark phase proceeded. Of the 480 time points, only 11 were significantly different between day 14 and day 35.

Although rats consuming HFD in 3 meals displayed a similar ramped cumulative food intake (Figure 2B) to that seen in study 1, the second (midnight) meal became progressively larger than the other meals (*P* < 0.01; Figure 2E). In addition, food consumption during the first 30 min of meal 2 was elevated by 45% between days 14 and 35 (Figure 2D; *P* < 0.0001), meal duration being unaffected (Figure 2F). Analysis of meal microstructure revealed that only 7 of the 480 time points were significantly different between days 14 and 35 (**Supplemental Figure 5**).

As with StD (study 1), the CLAMS output data were strongly correlated with the data derived from manual measurement of daily HFD intake (*R*^2^ = 0.89, all data; Figure 2G), with an overall accuracy of 98.4% ± 0.9%. Similar relations were observed for grazing-only and meal-fed-only animals (grazing: *R*^2^ = 0.86; accuracy: 99.0% ± 1.4%; meal-fed: *R*^2^ = 0.91; accuracy: 97.5% ± 1.4%).

As in study 1, when compared with rats consuming the HFD ad libitum, grazing and meal-fed rats showed a 17% and 15% reduction in total cumulative food intake (*P* < 0.01; Figure 2H), which became apparent from days 13 (grazing) and 6 (meal-fed) onwards. Again, cumulative food intake was not different between the grazing and meal-fed rats at any time during the 6-wk study. It should be noted that rats consuming the HFD ad libitum showed a transient hypophagia (in comparison to rats receiving an LFD) between days 3 and 18, but this initial ability to regulate caloric intake was eventually overcome, with ad libitum/HFD-fed rats consuming 112% of the cumulative caloric intake of ad libitum/LFD-fed rats by day 42 (*P* = 0.019; Figure 2H).

### Study 3: Do these meal patterns activate the stress axis?

To assess whether these feeding patterns activate the HPA axis, we performed automated serial blood sampling and quantification of corticosterone secretion in pattern-fed rats.

**FIGURE 3:**
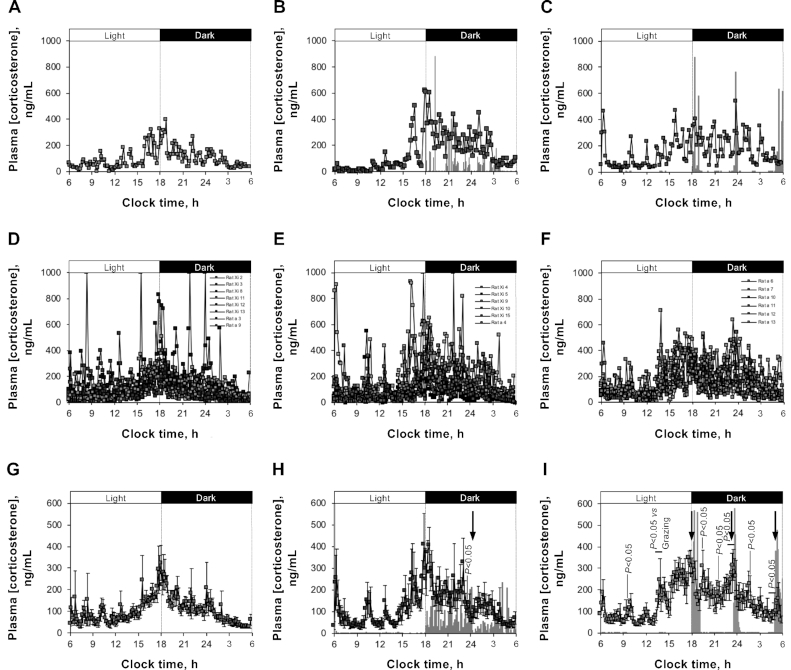
Effect of ad libitum, grazing, or meal feeding with StD on corticosterone secretion in male rats (study 3). Data shown are representative (A–C), superimposed (D–F), and mean ± SEM (G–I) 24-h corticosterone profiles from male Sprague-Dawley rats on day 20–21 of receiving StD in either ad libitum (A, D, G; *n* = 8), grazing (B, E, H; *n* = 6), or meal-fed (C, F, I; *n* = 6) patterns. Statistical comparisons were made by 1-factor ANOVA and Kruskal-Wallis and Dunn's multiple-comparison post-hoc tests (G–I); *P* < 0.05 compared with ad libitum–fed animals unless otherwise stated. Corresponding individual (B, C) and mean (H, I) feeding event data are shown in the background. Arrows show a coordinated decline in corticosterone concentration and feeding (H) and coordinated peaks of corticosterone secretion and feeding (I). StD, standard nonpurified rodent diet.

When compared with ad libitum feeding (**Figure 3**A, D, G), grazing had little effect on corticosterone secretion (Figure 3B, E, H), there being no change in total (AUC) or baseline (OC_5_) secretion (**Table 1**), circadian timing (Table 1; **Supplemental Figure 6**), peak height or nadir value, or the height or frequency of the ultradian pulses (Table 1) and little change in the mean profile (Figure 3H). However, the sharp coordinated decline in circulating corticosterone that occurred in the middle of the dark phase (2300–0010) preceded a temporary cessation in feeding (Figure 3H, arrow).

**TABLE 1:** Parameters of corticosterone secretion in male rats fed standard nonpurified rodent diet in either ad libitum, grazing, or meal-fed patterns (study 3)^1^

	Ad libitum	Grazing	Meal-fed
AUC, μg · mL^–1^ · min^–1^	151 ± 23^a^	182 ± 36^a^	219 ± 21^a^
Circadian peak time, min after 0600	741 ± 49^a^	808 ± 53^a^	821 ± 37^a^
Circadian peak value, ng/mL	169 ± 22^a^	205 ± 40^a^	244 ± 24^a^
Circadian nadir value, ng/mL	55.7 ± 8.3^a^	60.1 ± 11.6^a^	81.8 ± 11.3^a^
Baseline (OC_5_), ng/mL	10.6 ± 3.2^a^	15.5 ± 5.8^a,b^	36.3 ± 7.9^b^
Ultradian peak number, peak/24 h	23.5 ± 0.8^a^	21.2 ± 0.9^a^	21.7 ± 1.0^a^
Ultradian peak height, ng/mL	175 ± 30^a^	233 ± 43^a^	252 ± 19^a^
Ultradian peak mass, ng · mL^–1^ · min^–1^	210 ± 32^a^	264 ± 59^a^	265 ± 28^a^
Adrenal gland weight, mg	24.5 ± 1.6^a^	24.9 ± 2.0^a^	22.7 ± 1.7^a^
Cumulative food intake, g	542 ± 12^a^	482 ± 10^b^	462 ± 18^b^

1 Data presented are means ± SEMs [ad libitum (*n* = 8), grazing (*n* = 6), or meal-fed (*n* = 6)]. Statistical comparisons were made by 1-factor ANOVA and Bonferroni post-hoc tests; labeled means in a row without a common letter differ, *P* < 0.05. OC_5_, observed concentration 5 (the cutoff value below which 5% of the samples fall when ranked in ascending order of concentration).

In contrast, although the AUC was unaffected in meal-fed rats, baseline secretion (OC_5_) was trebled (Table 1), an effect particularly prominent in the period from 1200 to 1800 [Figure 3C, F; OC_5_ (ad libitum): 21.0 ± 2.7 ng/mL; OC_5_ (grazing): 20.0 ± 8.1 ng/mL; OC_5_ (meal-fed): 52.1 ± 7.8 ng/mL; *P* = 0.0101 (compared with ad libitum) and 0.0104 (compared with grazing)]. Although meal feeding did not alter circadian timing, peak height, or nadir value (Table 1; Supplemental Figure 6), or change the height or frequency of ultradian pulses (Table 1), calculation of mean profiles revealed the presence of coincident corticosterone pulses that were synchronized between individual meal-fed rats (Figure 3I). Six of these bursts were significantly higher than in ad libitum–fed rats (*P* < 0.05) and 2 were higher than in grazing rats (*P* < 0.05; Figure 3I).

In meal-fed rats each meal was associated with a preceding peak in corticosterone secretion, the commencement of feeding resulting in a sharp decline in circulating corticosterone (Figure 3I, arrows). However, other peaks in corticosterone secretion in meal-fed rats were not associated with temporal feeding events, the peaks occurring at ∼2-h intervals.

Despite these meal pattern–dependent changes in corticosterone secretion, adrenal gland weight was unaffected in either grazing or meal-fed rats (Table 1), even after more prolonged feeding with StD (study 1) or HFD (study 2) (data not shown).

### Study 4: Can grazing and meal feeding protocols be used in mice and are the effects seen Ghr dependent?

Because spontaneous feeding patterns are disturbed by deletion of the *ghrelin* receptor (26), we used our CLAMS system to deliver these 3 patterns of food intake to adult male Ghr^−/−^ mice and their WT littermates for 3 wk.

**FIGURE 4:**
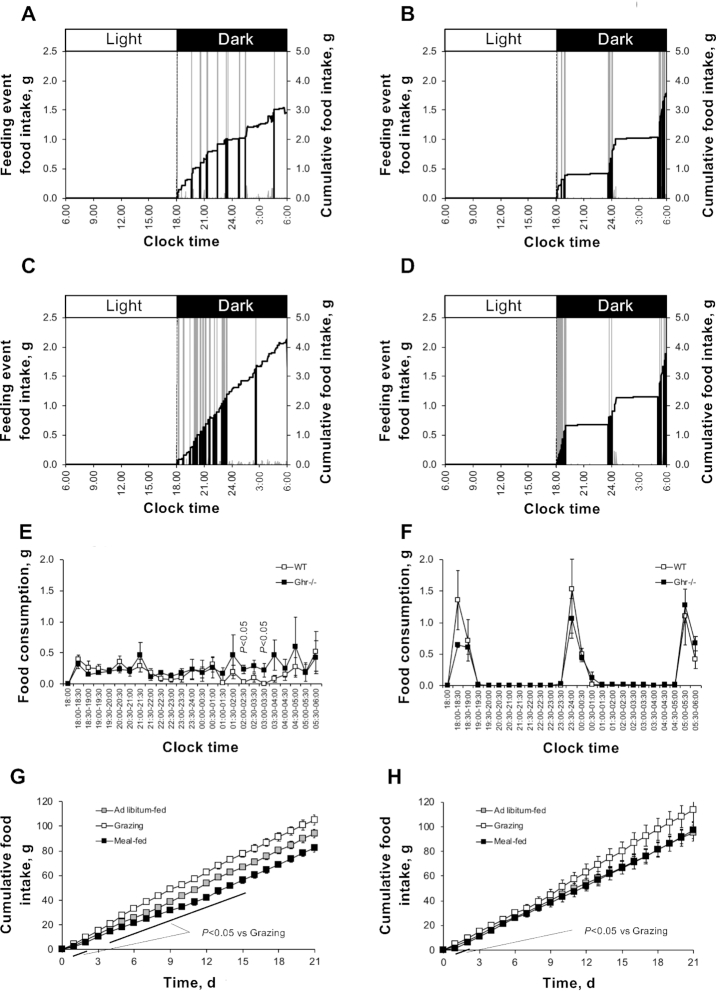
Delivery of StD in grazing and meal-feeding patterns in male WT and Ghr^−/−^ mice (study 4). Representative daily food intake profiles from day 14 of a 3-wk study in WT (A, B) and Ghr^−/−^ (C, D) mice fed StD (13.9% Atwater Fuel Energy fat) in either grazing (A, C) or meal-fed (B, D) patterns. Each profile shows cumulative food intake (black line; right-hand axes) superimposed onto the individual feeding events (gray bars; left-hand axes). Comparison of the mean day-14 grazing (E) and meal-fed (F) food consumption profiles in WT and Ghr^−/−^ mice is shown, with cumulative food intake across the 3-wk study in WT (G) and Ghr^−/−^ (H) mice presented. Data shown are either representative individual profiles (A–D) or means ± SEMs [E–H; *n* = 5 (grazing) and 6 (meal-fed)] with statistical comparisons performed by either unpaired Student's *t* test (E, F) or 1-factor ANOVA and Bonferroni's selected-pairs post-hoc test (G, H). Note: groups of ad libitum–fed WT and Ghr^−/−^ mice (G, H; *n* = 8 both groups) were monitored in parallel to calculate food intake allowances for application to grazing mice. Ghr^−/−^, *ghrelin* null; StD, standard nonpurified rodent diet; WT, wild-type.

It was immediately apparent that individual consumption profiles showed large negative/positive deflections (**Figure 4**A–D), resulting from mice stepping on/off the food hopper while feeding. As a result, the CLAMS-derived data were less well correlated with the manually measured food intake [*R*^2^ (all mice) = 0.43; regression analysis not shown]. To overcome these inaccuracies in the data, the large negative and positive displacement values were set to zero and the difference between these values entered into the next data point [e.g., for mouse Ω19 (Figure 4C; grazing Ghr^−/−^) the values −30.62, −0.38, +31.13, 0 (for time points 20.850, 20.875, 20.900, 20.925 h on day 14) were corrected to 0, 0, 0, +0.13]. Thus, although the feeding microstructure in the 90-s data was not analysed, the 30-min data remained correct (giving an accuracy of 106.9% ± 6.8% of manually measured daily food intake), thereby enabling further analysis.

The food consumption patterns produced in mice were similar to those produced in rats. Grazing WT mice consumed StD uniformly over the first 4 h of the dark phase, with a marked nadir in consumption from 0200 to 0400, rising towards the end of the dark phase (Figure 4A, E). In contrast, grazing Ghr^−/−^ mice showed a more consistent consumption pattern across the whole dark phase, being significantly higher than their WT littermates at 0200–0230 and 0300–0330 (*P* < 0.05) (Figure 4C, E).

Meal feeding produced 3 equally sized meals in WT mice (Figure 4B, F). Although total day-14 food consumption in Ghr^−/−^ mice was not significantly different from that in WT mice (Figure 4F), the total diet consumed in the first meal was 40% lower than in WT mice (*P* < 0.05; data not shown). Meals 1 and 3 in Ghr^−/−^ mice were also 34% and 17% longer in duration than the corresponding WT meals (*P* < 0.05; data not shown).

Cumulative food intake was 28% higher in grazing than meal-fed WT mice (Figure 4G), being significantly elevated from day 1 to day 15 (*P* < 0.05). When compared with the data obtained from the parallel study in rats (Figure 1H), it can be seen that this is due to the increase in permitted dietary intake in grazing mice (see Methods), rather than a reduction in meal-fed mice. This pattern-dependent difference in food intake was abolished in the absence of Ghr: meal-fed Ghr^−/−^ mice only consumed less than their grazing counterparts for the first 2 d of the study, (Figure 4H). Grazing had no effect on adrenal gland weight in either WT or Ghr^−/−^ mice (data not shown).

## Discussion

We present here a novel approach to establishing the physiologic impact of temporal feeding patterns. Our approach has several benefits.

First, our CLAMS-based system enables us to replicate relevant human feeding patterns (e.g., grazing and “3 meals a day”) in rodents. This is possible because the modified Oxymax control software permits the programming of multiple feeding events in which access is regulated by time and amount of food consumed. The delivery of 3 consistent meals is clearly an advance over the earlier reversed feeding (13, 14) or single-meal-feeding studies (17, 18), and by limiting these events to the dark phase, our approach overcomes the obesogenic influence of reversed feeding (14) or the necessity to acclimatize animals to a reversed-light protocol. Indeed, if required, the system can deliver different sizes, numbers, or frequencies of meals at any time of the light or dark phases.

However, the real advance is the prevention of large meal consumption. By presenting small quantities of diet every 30 min, we have produced a smoothed food consumption in which the contribution of meal feeding seen in ad libitum–fed animals is excluded and the stomach never filled to capacity. In addition, the use of crushed diet prevents food hoarding in the home cage, thereby excluding the confounding influence of uncontrolled consumption. The evidence that grazing and meal-fed rats consumed the same overall quantity of food was fortuitous, but enables any differences in biological outcome to be attributed to pattern per se. Had this outcome not occurred, the system could be programmed to pair either food or caloric intake between rodents receiving different patterns or diets.

Second, scanning the undercage balances every 90 s yielded a vast quantity of previously unattainable data, enabling more complex analysis of feeding microstructure, such as the frequency and consistency of individual feeding events within each 30-min timeframe.

Third, the coapplication of serial blood sampling in rats (study 3) confirms that this approach does not elevate overall corticosterone secretion, or disrupt circadian and ultradian rhythmicity. Given that the effectiveness of many metabolically active hormones, including Ghr (27, 28), growth hormone (35, 36), and corticosterone (37), is determined by their temporal pattern of secretion, the ability to perform serial blood sampling in pattern-fed animals enables assessment of the temporal relation between secretory and feeding events (see below).

Our approach is not without its drawbacks. Laboratory rodents are social creatures. Although transparent CLAMS cages permit visual and auditory communication, the use of single housing to monitor the food intake of individual animals restricts social interaction. This could be overcome by modifying a system designed to permit diet access to individually identified animals (38) or by through the use of paired cages in which animals are separated by a grid (if automated blood sampling was required). However, the absence of a rise in total corticosterone secretion (study 3) indicates that the HPA axis is not activated by the current approach.

Rats, in particular, take measures to maintain access to the diet (e.g., wedging environmental enrichment objects under the hopper lid), but this can be overcome by securing these items. Mice, on the other hand, are small enough to stand on the hopper, generating a “negative food intake” (study 4). Although this can be overcome through the use of mouse-specific cages, calculating the difference between “mouse on” and “mouse off” data yielded an overall consumption error (<7%) comparable to that in rats. It should be remembered that rodents are coprophagic and may consume fecal pellets outside of the controlled feeding times. Nevertheless, despite these procedural limitations, the difference between the recorded and manually measured food intake was <8%.

Last, the cage footprint of our current system is designed for use with rats weighing ≤500 g. Although these will accommodate most female rats, male Sprague-Dawley rats become too large beyond 10–12 wk of age, necessitating the construction of larger housing for studies in aged animals. Conversely, the rat system is too large to be used with juvenile mice as they may become trapped inside the food access system. Thus, we are currently restricted to studying rats with a body weight of <500 g and mice with a body weight of >30 g.

Despite these limitations, our study has yielded 2 important biological findings. First, although grazing and meal feeding reduced cumulative food intake in rats (studies 1 and 2) our mouse study (study 4) revealed that when the nocturnal grazing allowance was less restricted, grazing produces hyperphagia (compared with meal-fed mice). This difference in consumption appears to be mediated by the orexigenic hormone Ghr (25), because it was abolished in Ghr^−/−^ mice.

Interestingly, our data also imply that the removal of Ghr signaling also impairs both meal initiation and meal-induced satiety. Although grazing WT mice consumed most of their diet in the early phase of each time period, Ghr^−/−^ mice showed a less prominent feeding onset and a more consistent consumption profile across the whole dark phase (Figure 4E). Similarly, the absence of Ghr produced a smaller first meal in the meal-fed mice, with meals 1 and 3 being of longer duration. These findings corroborate evidence from Ghr-receptor^−/−^ mice, which consume larger spontaneous meals with a longer feeding duration (26). Thus, although Ghr signaling may be important for meal initiation, the marked reduction of Ghr in obesity (39) may be a contributory factor in prolonged hyperphagia. Thus, the temporal dynamics of signaling is important.

Whether feeding patterns affect other Ghr-dependent metabolic processes remains to be determined, but our data imply that although the contemporary shift in feeding behavior from regular meals towards grazing (4) may initially increase satiety (studies 1 and 2) (40, 41), this pattern of food intake may actually result in overconsumption when food availability is less restricted.

Second, although overall corticosterone secretion and ultradian pulse amplitude were unaffected, meal-feeding induced temporal changes in HPA axis activity, including a marked elevation in baseline secretion prior to the commencement of the first meal and temporal alignment of secretory peaks between individual animals (study 3). The ultradian corticosterone peaks are thought to be generated by the intrinsic feedforward/feedback activity of the axis, the frequency being determined largely by the delay in de novo synthesis of corticosterone in the adrenal cortex (37). Ad libitum–fed rats receive 1 major environmental cue, the light-dark cycle, which controls the circadian secretion of corticosterone (42). Grazing rats receive 2 coincident cues (lights-off reinforced by feeding commencement) and again corticosterone secretion remains unaltered. In contrast, meal-fed rats receive the same reinforced cue at lights-off, plus 2 subsequent temporal cues (meals 2 and 3), each of which is associated with a concurrent surge in corticosterone secretion. Although this has no significant effect on circadian timing, it is possible that these additional time-locked events act to phase-reset ultradian activity, as occurs with acute psychological stimulation (43), the influence of this additional triggering enduring into the nonfeeding light phase. These effects aside, the absence of any change in the overall ultradian rhythmicity of corticosterone indicates that glucocorticoid receptor activation is likely to be maintained (37) with both grazing and meal feeding.

Thus, it can be postulated that, because hormone pulsatility is a major determinant of biological activity, its triggering by patterned feeding is likely to be important in mediating the potential impact of temporal feeding patterns on a wide range of physiologic processes. These could include the metabolic, endocrine and neurogenic actions of Ghr (16, 27, 28), the skeletal and metabolic impact of growth hormone (35, 36), and the influence of corticosterone on appetite control and learning and memory (37, 44).

In summary, we report here a novel approach that has enabled the maintenance of laboratory rodents on consistent, measurable, researcher-defined, stress-free, temporal feeding patterns. This long-overdue advance will facilitate rapid progress in resolving a major unanswered question in nutritional science: what is the physiologic impact of ultradian feeding patterns? Indeed, given that the biological action of many metabolic hormones is itself dependent upon the temporal pattern of tissue exposure, our coapplication of automated serial blood sampling elevates the study of mechanistic temporal dynamics in chrononutrition to a new level of sophistication.

## Supplementary Material

nxz116_Supplement_Files

## Notes

Supported by the Waterloo Foundation (grant 08-BM058R to TW); the Bill & Melinda Gates Foundation (grant OPP1061040 to TW); the Endocrine Society (Summer Research Fellowship to TWT); Cardiff University's NMHRI Seedcorn fund (grant TWDD2016 to TW); the Foundation for Prader-Willi Research (grant FPWRTW052011 to TW); the Medical Research Council (fellowship MR/N008936/1 to JJW); the Wellcome Trust (grant WT105618MA to JJW); and the Engineering and Physical Sciences Research Council (grant EP/N014391/1 to JJW).

Author disclosures: TWT, RDB, MJW, BA-W, JJW, YS, and TW, no conflicts of interest.

Supplemental Figures 1–6 and Supplemental Table 1 are available from the “Supplementary data” link in the online posting of the article and from the same link in the online table of contents at https://academic.oup.com/jn/.

Abbreviations used: AFE, Atwater Fuel Energy; CLAMS, Comprehensive Laboratory Animal Monitoring System; Ghr, ghrelin; HFD, high-fat diet; HPA, hypothalamopituitary adrenal; LFD, low-fat diet; OC_5_, observed concentration 5 (the cutoff value below which 5% of the samples fall when ranked in ascending order of concentration); StD, standard (nonpurified) rodent diet; tdF/I, total daily food intake; WT, wild-type.
