# Supplementary material for: A Novel Automated System Yields Reproducible Temporal Feeding Patterns in Laboratory Rodents
Source: J Nutr. 2019 Jul 9;149(9):1674–84. doi: 10.1093/jn/nxz116 (PMC6736427; doi:10.1093/jn/nxz116)
Supplement: nxz116_Supplement_Files [file nxz116_supplement_files.zip › Fig S1 - CLAMS System.pdf]

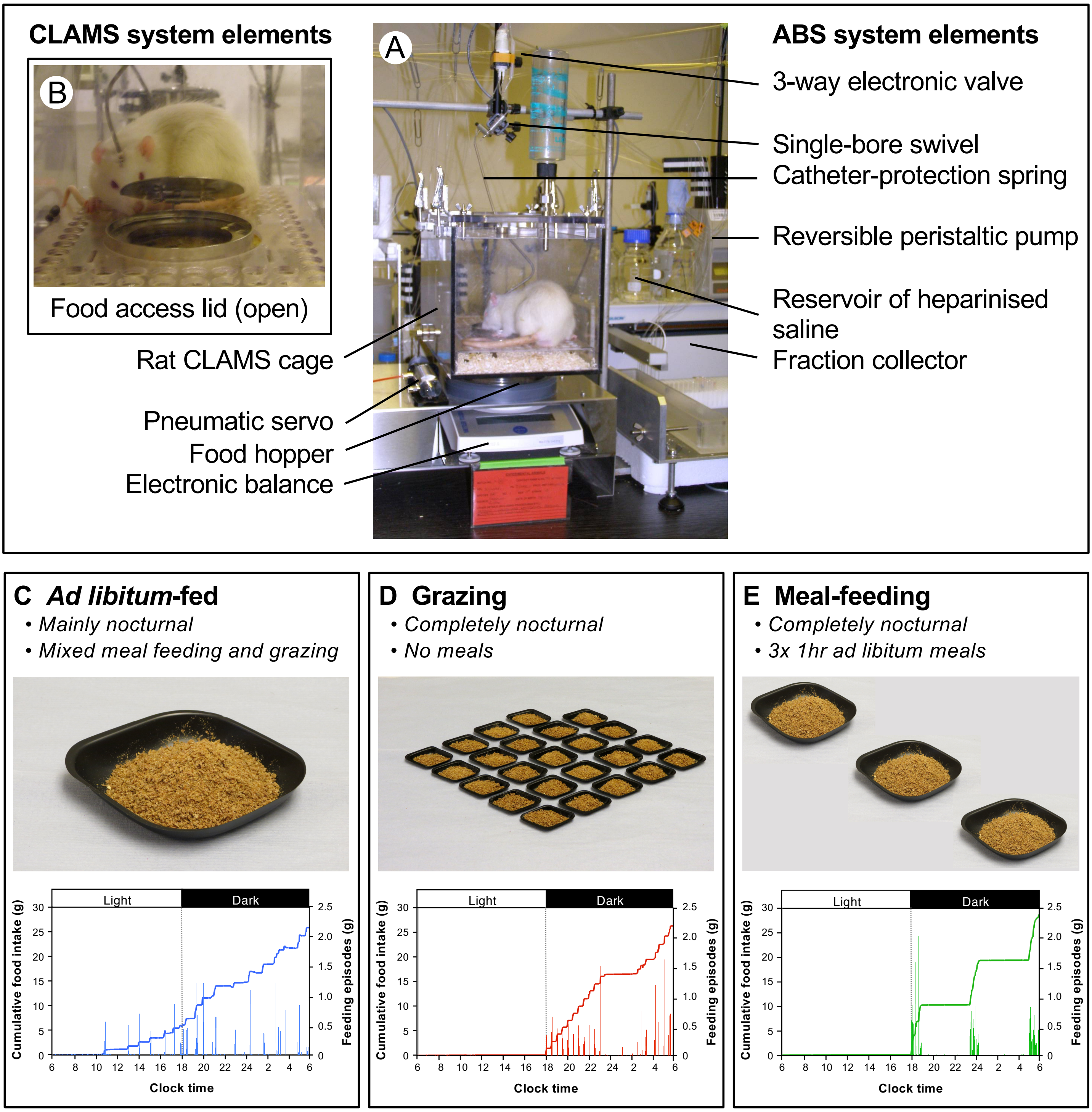

Figure S1: An automated feeding and blood sampling system. A computer-controlled CLAMS-based system (A) including individual rat cages and an under-floor food hopper located on a balance used to regulate the position of a food access lid (B) in terms of time and the amount of food consumed. This enables delivery of crushed diet in either a nocturnal grazing pattern (D; providing one 24th of the total daily food intake of *ad libitum*-fed rats (C) every 30 mins) or in meals (E; three 1h meals per night). A computer-controlled automated blood sampling (ABS) system (A) was used to assess the impact of temporal feeding patterns on the stress axis.
