# Supplementary material for: A Novel Automated System Yields Reproducible Temporal Feeding Patterns in Laboratory Rodents
Source: J Nutr. 2019 Jul 9;149(9):1674–84. doi: 10.1093/jn/nxz116 (PMC6736427; doi:10.1093/jn/nxz116)
Supplement: nxz116_Supplement_Files [file nxz116_supplement_files.zip › Fig S3 - Meal Periods.pdf]

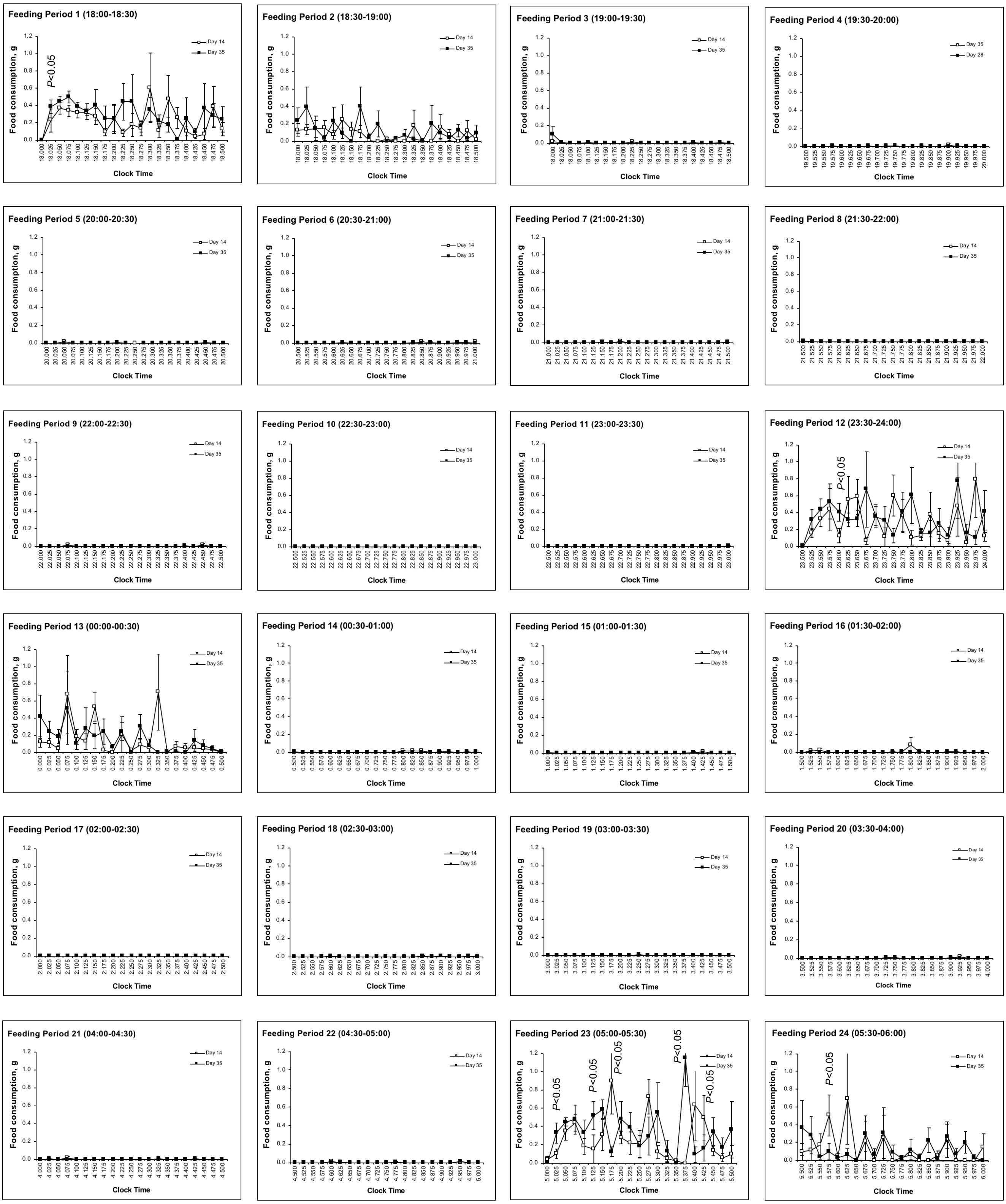

Figure S3: Consistency of individual feeding periods in male rats meal-fed with a standard non-purified rodent diet (Study 1). A comparison between day 14 and day 35 of all 24 30-minute feeding periods during the dark phase in meal-fed male Sprague-Dawley rats. Data shown are mean ± SEM (*n*=5 for both days), with statistical comparisons of individual timepoints made by paired Student's t-test.
