# Supplementary material for: A Novel Automated System Yields Reproducible Temporal Feeding Patterns in Laboratory Rodents
Source: J Nutr. 2019 Jul 9;149(9):1674–84. doi: 10.1093/jn/nxz116 (PMC6736427; doi:10.1093/jn/nxz116)
Supplement: nxz116_Supplement_Files [file nxz116_supplement_files.zip › Fig S6 - Corticosterone profiles.pdf]

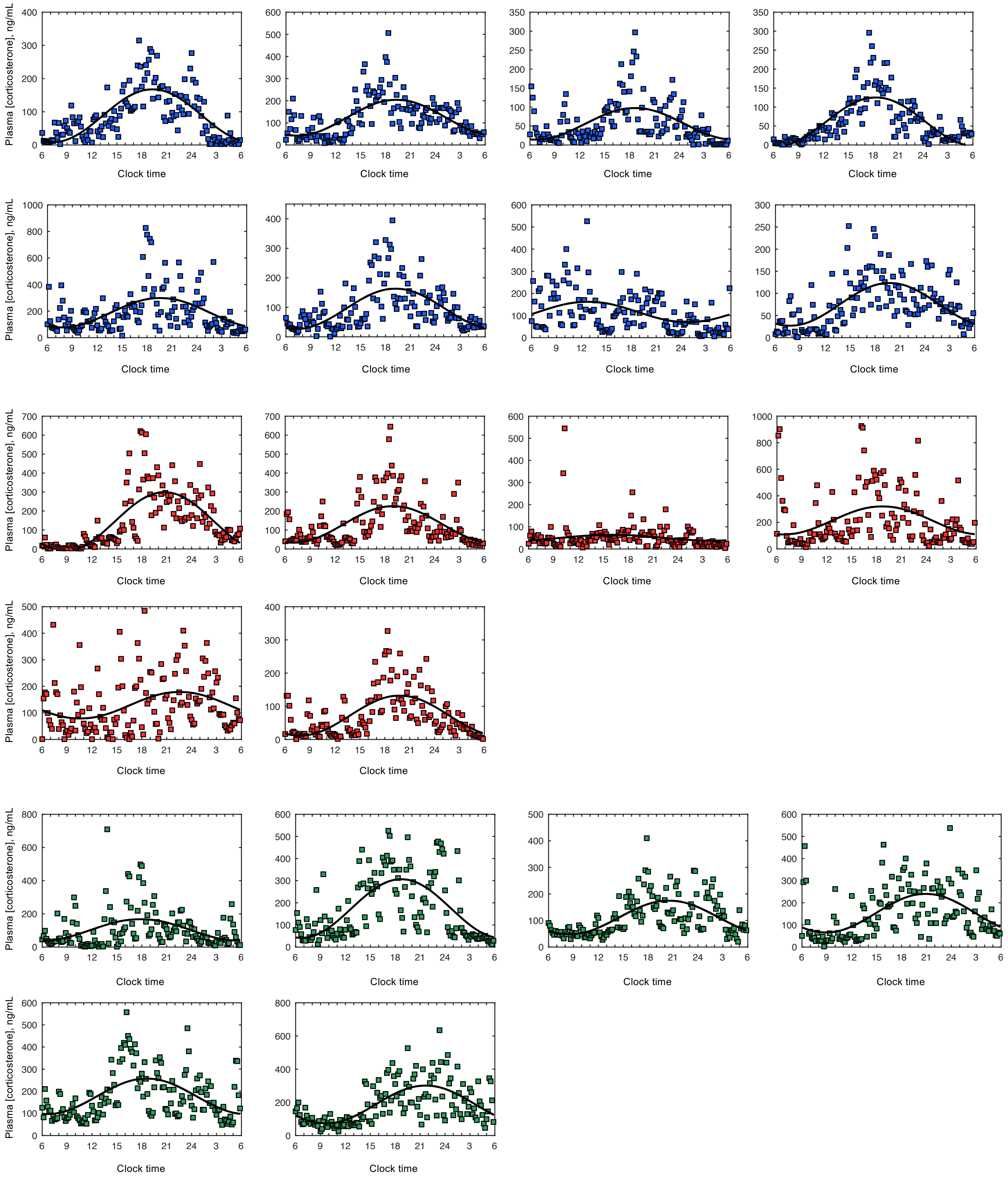

Figure S6: The effect of *ad libitum* (blue), grazing (red) or meal feeding (green) with a standard non-purified rodent diet on individual corticosterone profiles in male rats (Study 3). Sprague-Dawley rats received standard non-purified rodent diet for 3 weeks, with blood samples collected by automated serial sampling on day 20-21 after preparation with jugular vein catheters on day 18. Black curves show circadian sine function fitted to the data. Dark phase is 18:00-06:00h.
