# Supplementary material for: A Novel Automated System Yields Reproducible Temporal Feeding Patterns in Laboratory Rodents
Source: J Nutr. 2019 Jul 9;149(9):1674–84. doi: 10.1093/jn/nxz116 (PMC6736427; doi:10.1093/jn/nxz116)
Supplement: nxz116_Supplement_Files [file nxz116_supplement_files.zip › Supplementary Table S1.docx]

**Supplementary Data** Tilston TW et al, 2019; Supplemental Table 1

**Table S1: Dietary compositions**

|  | **Diets** | | |
| --- | --- | --- | --- |
|  | **SDS RM3 (E)**  Cat # 801066 | **SDS LFD**  Cat # 824040 | **SDS HFD**  Cat # 824043 |
| Nutrients, %  Moisture  Crude oil/fat  Crude protein  Crude fibre  Ash  Carbohydrate  Nitrogen-free extract | 10.0  4.25  22.39  4.21  7.56  55.7  51.20 | 4.22  3.97  18.71  3.82  3.82  64.3  64.17 | 4.51  22.56  23.04  4.60  4.45  39.8  39.65 |
| Energy  Gross energy, MJ/kg  Digestible energy, MJ/kg  Metabolisable energy, MJ/kg  Atwater fuel energy (AFE), MJ/kg  AFE from oil/fat, %  AFE from protein, %  AFE from carbohydrate, % | 15.21  12.42  11.36  13.90  11.50  26.93  61.57 | 16.25  14.81  13.44  15.38  10.0  20.0  70.0 | 20.21  18.45  17.03  18.97  45.0  20.0  35.0 |
| Fatty Acids, %  Saturated fatty acids  C12:0 Lauric  C14:0 Myristic  C16:0 Palmitic  C18:0 Stearic  Monounsaturated fatty acids  C14:1 Myristoleic  C16:1 Palmitoleic  C18:0 Oleic  Polyunsaturated fatty acids  C18:2 Linoleic  C19:3 Linolenic  C20:4 Arachidonic  C22:5 Clupanodonic | 0.05  0.20  0.36  0.09  0.01  0.13  1.03  1.15  0.17  0.22  0.04 | 0.02  0.03  0.39  0.15  0.01  0.02  0.80  1.87  0.26  0.01  0.00 | 0.03  0.31  4.28  1.91  0.02  0.03  6.43  3.75  0.39  0.01  0.00 |
| Amino Acids, %  Arginine  Lysine  Methionine  Cystine  Tryptophan  Histidine  Threonine  Isoleucine  Leucine  Phenylalanine  Valine  Tyrosine  Glycine  Aspartic acid | 1.54  1.33  0.34  0.34  0.27  0.57  0.86  0.98  1.68  1.03  1.10  0.80  1.88  1.43 | 0.59  1.18  0.46  0.37  0.17  0.43  0.65  0.94  1.42  0.78  1.13  0.78  0.74  1.05 | 0.73  1.46  0.57  0.45  0.20  0.53  0.80  1.16  1.75  0.96  1.39  0.96  0.91  1.30 |
| Macro Minerals, %  Calcium  Total phosphorous  Sodium  Chloride  Potassium  Magnesium | 1.15  0.82  0.32  0.43  0.81  0.29 | 0.49  0.19  0.13  0.22  0.36  0.06 | 0.63  0.37  0.16  0.28  0.45  0.08 |
| Micro Minerals, mg/kg  Iron  Copper  Manganese  Zinc  Cobalt  Iodine  Selenium  Fluorine | 188.17  20.28  102.01  51.34  0.617  1.395  0.498  9.24 | 49.01  7.10  11.22  55.89  0.00  0.207  0.160  1.01 | 59.90  8.76  13.84  68.97  0.00  0.255  0.197  1.24 |
| Vitamins  β-Carotene, mg/kg  Retinol, µg/kg  Vitamin A, iu/kg  Cholecalciferol, µg.kg  Vitamin D, iu/kg  α-Tocophenol, mg/kg  Vitamin E, iu/kg  Vitamin B_1_ (Thiamine), mg/kg  Vitamin B_2_ (Riiboflavin), mg/kg  Vitamin B_6_ (Pyridoxine), mg/kg  Vitamin B_12_ (Cyanocobalamine), µg/kg  Vitamin C (Ascorbic acid), mg/kg  Vitamin K (Menadione), mg/kg  Folic acid (Vitamin B_9_), mg/kg  Nicotinic acid (Vitamin PP), mg/kg  Pantothenic acid (Vitamin B_3/5_), mg/kg  Choline (Vitamin B_6/7_), mg/kg  Inositol, mg/kg  Biotin (Vitamin H), µg/kg | 0.15  5977  19924  102.2  4088.7  100.35  110.39  27.08  10.60  19.54  26.78  1.33  4.15  2.73  85.00  40.27  1641.65  1903.20  322.87 | 1200  4000  57.25  1000.00  71.03  79.0  4.95  5.17  5.79  25.00  0.00  0.77  1.99  29.70  14.61  1186.92  0.00  200.00 | 1480  4936  195.9  1417  90.59  100.72  6.10  6.37  7.15  30.85  0.00  0.95  2.38  36.65  18.48  1392.58  0.00  246.82 |

**Please note:** These values supplied by Special Diet Services are theoretical calculated values and are intended as a guide only. Levels of nutrients in the finished diet may be affected by natural variation of raw materials.
